# Development and evolution of the unique cetacean dentition

**DOI:** 10.7717/peerj.24

**Published:** 2013-02-19

**Authors:** Brooke A. Armfield, Zhengui Zheng, Sunil Bajpai, Christopher J. Vinyard, JGM Thewissen

**Affiliations:** 1 Department of Anatomy and Neurobiology, Northeast Ohio Medical University, Rootstown, Ohio, United States; 2 Howard Hughes Medical Institute and Department of Molecular Genetics and Microbiology, University of Florida, Gainesville, Florida, USA; 3 Department of Earth Sciences, Indian Institute of Technology, Roorkee, India; 4 Birbal Sahni Institute of Palaeobotany, Lucknow, UP, India

**Keywords:** Cetacean, Teeth, Evo-devo, Paleontology, Cetacea

## Abstract

The evolutionary success of mammals is rooted in their high metabolic rate. A high metabolic rate is sustainable thanks to efficient food processing and that in turn is facilitated by precise occlusion of the teeth and the acquisition of rhythmic mastication. These major evolutionary innovations characterize most members of the Class Mammalia. Cetaceans are one of the few groups of mammals in which precise occlusion has been secondarily lost. Most toothed whales have an increased number of simple crowned teeth that are similar along the tooth row. Evolution toward these specializations began immediately after the time cetaceans transitioned from terrestrial-to-marine environments. The fossil record documents the critical aspects of occlusal evolution of cetaceans, and allows us to pinpoint the evolutionary timing of the macroevolutionary events leading to their unusual dental morphology among mammals. The developmental controls of tooth differentiation and tooth number have been studied in a few mammalian clades, but nothing is known about how these controls differ between cetaceans and mammals that retain functional occlusion. Here we show that pigs, a cetacean relative with regionalized tooth morphology and complex tooth crowns, retain the typical mammalian gene expression patterns that control early tooth differentiation, expressing *Bmp4* in the rostral (mesial, anterior) domain of the jaw, and *Fgf8* caudally (distal, posterior). By contrast, dolphins have lost these regional differences in dental morphology and the *Bmp4* domain is extended into the caudal region of the developing jaw. We hypothesize that the functional constraints underlying mammalian occlusion have been released in cetaceans, facilitating changes in the genetic control of early dental development. Such major developmental changes drive morphological evolution and are correlated with major shifts in diet and food processing during cetacean evolution.

## Introduction

Most non-mammalian vertebrates have dentitions with simple tooth crowns, a single morphological tooth class (homodonty), and varying numbers of teeth per jaw (Gregory, 1922). In contrast, mammals have diverged from this state: their pleisomorphic dental pattern consists of four morphologically-distinct tooth classes (incisors, canine, premolars and molars) per jaw quadrant and a limited number of teeth erupting during a lifetime (Gregory, 1922). The evolution of multicuspid teeth and stable tooth number in the ancestors of mammals allowed for precise occlusion and hence the maintenance of a high metabolic rate through efficient food processing during mastication. These changes in dental patterns contributed significantly to the success of mammals and have been maintained in most species since the origin of mammals 220 million years ago (Luo, 2007).

While most mammals maintain some characteristics of this pleisomorphic dentition, a few species, such as phocid seals, armadillos, and cetaceans, have lost several characteristic mammalian dental traits along with precise dental occlusion. For example, in grey seals (*Halichoerus grypus*) the postcanine teeth act primarily in holding and puncturing prey (Adam & Berta, 2002). The morphology of their post-canine teeth reflect this shift away from precise occlusion and rhythmic mastication by showing a reduced number of mesial-distally arranged tooth cusps lacking tightly integrated occlusal surfaces (Miller et al., 2007; Jernvall, 2000). Interestingly, up to 10% of these seals also have supernumerary teeth; a much higher rate of anomalies than most mammals (Cruwys & Friday, 2006). The giant armadillo (*Priodontes maximus*) exhibits polydonty, or a significantly increased tooth number (approximately 20 per jaw quadrant) beyond the basal condition for placental mammals (3 incisors, one canine, 4 premolars, and 3 molars per quadrant, 3.1.4.3). Its teeth are homodont, unicuspid and did not occlude precisely.

**Figure 1:**
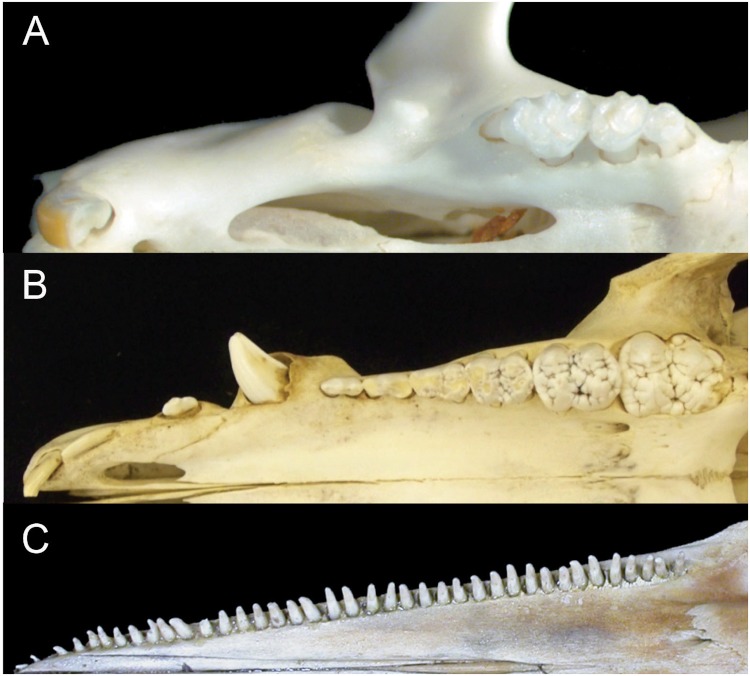
Comparison of mammalian dental patterns showing the differences in regionalization of tooth morphology. (A) *Mus musculus* (B) *Sus scrofa* (picture is of an immature pig with an unerupted M3) and (C) *Stenella attenuata*.

The only large radiation of mammals to lose precise dental occlusion is the order Cetacea. The absence of precise occlusion in cetaceans is associated with the evolution of both homodonty and polydonty (Fig. 1). It has been proposed that these profound changes in dentition are related to the absence of mastication in cetaceans (Werth, 2000). Cetaceans use their teeth to grab and hold, but not to chew their food. Homodonty and polydonty are acquired in cetacean evolution gradually, and even in mysticete evolution polydonty precedes their complete loss of teeth in modern forms. Monophyodonty, the presence of a single tooth generation, also characterizes cetaceans. This is unlike most other mammals (Nievelt & Smith, 2005), and appeared near the origin (Uhen, 2000) of the suborders Odontoceti (toothed whales, which includes dolphins) and Mysticeti (baleen whales).

The combination of these patterns makes it impossible to identify homologies among teeth (i.e. whether a tooth is a molar, premolar, canine or incisor). Given the correlated evolutionary changes among these morphological features, we hypothesize that crown complexity, homodonty and polydonty are developmentally related in these mammals with specialized dentitions.

Our understanding of how morphologically-distinct tooth classes and complex tooth crown shapes develop in mammals is largely derived from research on mice (Munne et al., 2010; Ohazama et al., 2010a; Ohazama et al., 2010b). The first morphological sign of tooth development begins with a thickening of oral epithelium along the maxillary and mandibular arches early in the embryonic period before individual teeth can be distinguished (Leche, 1892; Leche, 1895; Orban, 1980; Ruch et al., 1984). During this developmental stage, functional experiments on mice indicate that gene expression in the oral epithelium is crucial for controlling the shape and hence tooth class of individual teeth (Kollar & Baird, 1969; Sharpe, 1995; Tucker & Sharpe, 1999; Cobourne & Sharpe, 2003; Catón & Tucker, 2009; Ohazama et al., 2010b). Distinct gene networks are expressed in regions along the oral epithelium and induce areas to form either incisor teeth with simple, unicuspid crowns or more complex, multicuspid occlusal patterns typical of molar teeth. Specifically, bone morphogenetic protein, *Bmp4*, is expressed rostrally in the oral epithelium and initiates the signaling cascade that forms simple, single-cusped incisors (Vainio et al., 1993; Neubuser et al., 1997; Tucker, Matthews & Sharpe, 1998), whereas fibroblast growth factor *Fgf8*, mediates the formation of multicuspid molars in the caudal region of the oral epithelium (Sharpe, 1995; Tucker & Sharpe, 2004; Mitsiadis & Smith, 2006). Functional studies have also shown that changes in the location where these proteins are expressed along the tooth row leads to changes in tooth morphology and number. For example, ectopic *Bmp4* expression caudally, induced by the lack of a *Bmp4* antagonist, leads to formation of additional teeth with simplified crowns (Kassai et al., 2005; Murashima-Suginami et al., 2007; Munne et al., 2009). The expression patterns of molecular signals *Bmp4* and *Fgf8* regulate the signaling pathways that define whether a developing mouse tooth will be an incisor or molar, respectively.

Mice only have two tooth classes (incisors and molars, 1.0.0.3), but evidence from shrews which retain the four original mammalian tooth classes (Ogi et al., 2002) suggests that similar genetic mechanisms pattern their teeth. Nothing is known, however, about the initial protein signaling during the development of teeth in cetaceans or their terrestrial artiodactyl relatives (Nikaido, Rooney & Okada, 1999; Geisler & Theodor, 2009; Spaulding, O’Leary & Gatesy, 2009). Interestingly, some artiodactyls maintain the pleisomorphic mammalian dental pattern for tooth class and number and provide a compelling comparative group for identifying derived aspects of dental developmental in cetaceans (Fig. 1). Here we test the hypothesis that the pattern of early gene expression, presumably establishing heterodonty in developing mouse dentitions, can also be found in the pig (*Sus scrofa*) – a representative artiodactyl retaining four tooth classes, and a generalized dental formula (3.1.4.3). In addition, we test whether gene and protein expression in the developing dental lamina of pigs is similar to that in pantropical spotted dolphins (*Stenella attenuata*) which exhibit a simple crown morphology, homodonty and polydonty (more than 30 teeth per quadrant). Given our hypothesis that reduced crown complexity, homodonty and polydonty are genetically linked, we predict that protein expression during early dental developmental differs in dolphins from pigs, mice and shrews. To better understand the evolution of simplified crown morphologies, homodonty and polydonty in toothed whales, we examine gene (protein) expression during early dental development in dolphins and track morphological changes in tooth shape across early cetacean evolution.

## Materials and Methods

### Embryos

Pig embryos were collected from timed pregnancy sows from Ohio breeders. The embryos were immediately fixed in 4% paraformaldehyde in 1 × PBS overnight at 4 °C. They were dehydrated in a methanol series: 25%, 50%, 75%, 100% methanol/ 1 × PBS and stored at −20 °C. The pantropical spotted dolphin embryos are part of a museum collection at Natural History Museum of Los Angeles County (LACM). They were transferred for histological, cross-sectional study to Thewissen, and are on long-term loan to NEOMED, but will eventually be returned to LACM. Pig specimens are also in the NEOMED collection. Specimens are described in Table 1. They were fixed in 10% formalin buffer and stored in 70% ethanol after collection. Prior to use the embryos were aged according to Carnegie stages (Thewissen & Heyning, 2007). Due to the limited supply of dolphin embryos, we were only able to stain one specimen at each stage. Mice (C57/B6strain) were mated overnight and the morning of a vaginal plug was regarded as embryonic day E0.5. Embryos were harvested at E10.5, 11.5 and processed following the same protocol as for pigs.

**Table 1:** Embryonic Specimens Studied. Abbreviations – CA, embryonic Carnegie stage; E, embryonic day; IHC, Immunohistochemisty; In situ, in situ hybridization; NEOMED, Northeast Ohio Medical University; LACM, Natural History Museum of Los Angeles County.

Collection	Number	Species	Stage	Method and result	Number of micr. slides	Data presented
LACM	94594	Dolphin	Ca 14	IHC no relevant staining	271	
LACM	94617	Dolphin	Ca 14	IHC no relevant staining	141	
LACM	94747	Dolphin	Ca 16	IHC no relevant staining	324	
LACM	95670	Dolphin	Ca 17	IHC BMP4 and FGF8	297 (head)	Fig. 5
LACM	95634	Dolphin	Ca 18	IHC no relevant staining	402	
LACM	95817	Dolphin	Ca 19	IHC no relevant staining	513 (head)	
LACM	95671	Dolphin	Ca 20	IHC no relevant staining	247	
LACM	94650	Dolphin	Ca 17	3D of dental lamina	499 (head)	Fig. 4
NEOMED	P206	Pig	E21	in situ BMP4		Fig. 3
NEOMED	P214	Pig	E21	in situ FGF8		Fig. 3
NEOMED	P130	Pig	E22	in situ BMP4		
NEOMED	P155	Pig	E22	in situ FGF8		
NEOMED	P105	Pig	E24	in situ BMP4		
NEOMED	P107	Pig	E24	in situ FGF8		
NEOMED	P206	Pig	E21	IHC BMP4 and FGF8	35	
NEOMED	P6005	Pig	E21	IHC BMP4 and FGF8	104	
NEOMED	P6014	Pig	E21	IHC BMP4 and FGF8	124	
NEOMED	P112	Pig	E21	IHC BMP4 and FGF8	289	
NEOMED	P111	Pig	E21	IHC BMP4 and FGF8	282	
NEOMED	P007	Pig	E21	IHC BMP4 and FGF8	100	

### Histology and immunohistochemical staining

To determine protein signaling during tooth development, we assessed the location of BMP4 (Santa Cruz-6896) and FGF8 (Santa Cruz-6958) in the developing dental lamina by immunohistochemistry. Dolphin embryos were dehydrated to 100% ethanol, washed in several xylene baths and embedded in paraffin. The paraffin blocks were sectioned at 7 µm. After sections were rehydrated, the slides underwent heat-induced antigen retrieval using 0.01 M sodium citrate pH 6.0 to reverse the cross-linking between the proteins in the tissue and formalin. This was carried out for 10 min at 90–100 °C with 20 min incubation. To prevent enzyme activity naturally present in tissues from reacting with the DAB (Diaminobenzidine), an additional step of 3% hydrogen peroxide was applied to the slides for 5 min.

To detect the proteins of interest, we used the avidin–biotin complex method as this approach increases the sensitivity of tagging the protein of interest. We followed the protocol using the goat ABC kit (Santa Cruz-2023). Optimization of concentration experiments were performed for each antibody and both antibodies were optimized at a concentration of 1:500 bovine serum. The sections were counterstained with 0.01% Thionin for 15 s.

Positive staining was determined by the presence of DAB within the tissue. To ensure positive staining was not exogenous, we used two control methods. The first is a negative control that performed the ABC kit protocol without adding the primary antibody to the control slide. The second was a positive control. We compared protein expression across mice, pigs and dolphins of similar ages in tissues outside of the dental regions to determine if the patterns were similar. Positive controls were also compared to literature sources that indicate the proteins of interest should be present in certain tissues during the developmental stages analyzed.

We used immunohistochemistry to document protein localization in our dolphin embryos, as RNA is not preserved in our samples, and legal and ethical issues preclude the acquisition of fresh dolphin embryos.

### *In situ* hybridization

To ensure protein localization was similar to RNA expression in the pigs, we performed *in situ* hybridization on similar-aged pig embryos based on stage of dental development. Pig-specific probes were cloned from pig cDNA collected on gestation day 21. RNA was extracted using trizol. Polymerase chain reaction was performed to amplify fragments of the pig *Bmp4* and *Fgf8* gene using primers synthesized to amino acid sequences conserved across known mammals and found in pig ESTs. For *Bmp4* the forward primer TTA ACC TCA GCA GCA TCC CAG A and the reverse primer ACA ATC CAG TCA TTC CAG CCC A were used to amplify a 556bp fragment. For *Fgf8* the forward primer TGC TGT TGC ACT TGC TGG TTC T and the reverse primer ATG CAG ATG TAG AGG CCC GTT T were used to amplify a 298bp fragment. PCR amplifications were carried out with a melting temperature of 94 °C for 1 min, 58 °C annealing temperature for 30 s and 72 °C for 1 min extensions for 38 cycles and held at 72 °C for 10 min. PCR products were gel extracted and reamplified using the same PCR protocol. That PCR product was subcloned into the pCR2.1-TOPO TA vector (Invitrogen). Digoxygenin-UTP labeled probes were made following Roche DIG RNA Labeling kit (11175025910).

Whole-mount *in situ* hybridization (ISH) was performed according to Acloque, Wilkinson & Nieto (2008). To digest tissue and increase probe penetration a proteinase K (10 µg/ml) treatment was applied for 5 min, followed by 1 × PBS with tween-20 washes. Probe was added at a concentration of 0.5 µl/ml of prehybridization solution for 48 h at 65 °C. Localization of the probe is detected with an alkaline phosphate-conjugated anti-DIG antibody (Antidig-AP Fab Roche 11093274910) and 5-bromo-4-chloro-3-indolyl-phosphate (BCIP) plus 4-nitro blue tetrazolium chloride (NBT).

### Cetacean dental evolution

**Figure 2:**
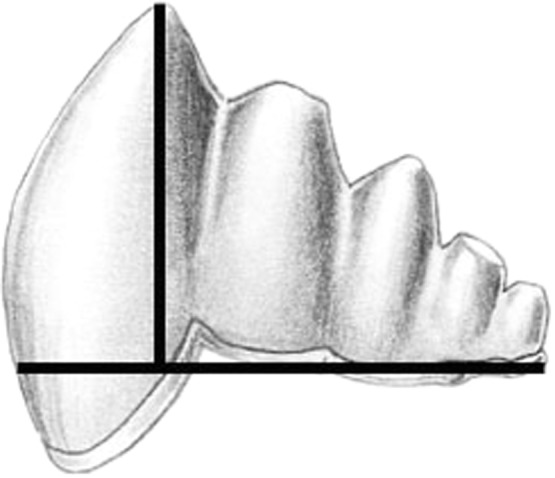
Cusp pointedness measurement. This value was quantified as cusp height divided by tooth length as shown on a basilosaurid lower molar.

We documented morphological changes in the dentition of fossil and modern Cetacea using our own collections and supplemented with data from the literature (Table 1). We focused on fossils from the Eocene, when cetaceans strongly reduced precise occlusion (Thewissen et al., 2011) and the Oligocene, when polydonty and homodonty originated in both odontocetes (Fordyce, 2003) and mysticetes (Deméré et al., 2008). In mysticetes, these changes preceded the loss of teeth altogether (Deméré et al., 2008). As part of this morphological analysis, we quantified pointedness as cusp height divided by tooth length (see Salazar-Ciudad & Jernvall, 2010) for lower molars of both fossil and modern species (Fig. 2). Cusp height was measured as the perpendicular distance from the cusp tip to a line connecting the rostral and caudal enamel–dentin junctions. Molar length is measured as the distance between the rostral and caudal enamel–dentin junctions. Larger values represent increased pointedness and document the morphological shift toward a simple, single-cusped tooth crown typical of many living odontocetes.

**Figure 3:**
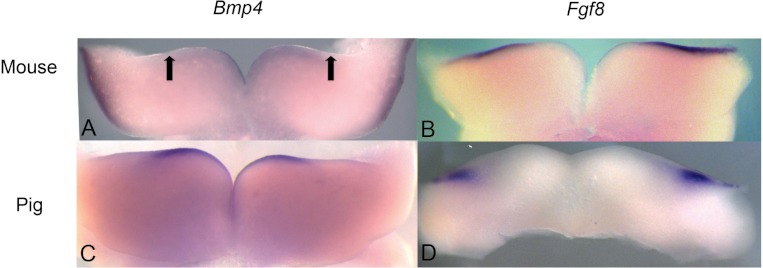
Comparison of *Bmp4* and *Fgf8* expression in mouse and pig using *in situ* hybridization. (A) *Bmp4* expression in E11.5 lower mouse developing jaw. Expression is found in the anterior portion of the oral epithelium, between the arrows. (B) *Fgf8* expression in E11.5 lower mouse developing jaw. Expression is found in the posterior portion of the oral epithelium (C) *Bmp4* expression in E21 lower pig developing jaw (NEOMED P206). Expression is found in the anterior portion of the oral epithelium similar to that seen in the mouse (D) *Fgf8* expression in E21 lower pig developing jaw (NEOMED P214). Expression is found in the posterior portion of the oral epithelium similar to that seen in the mouse.

**Figure 4:**
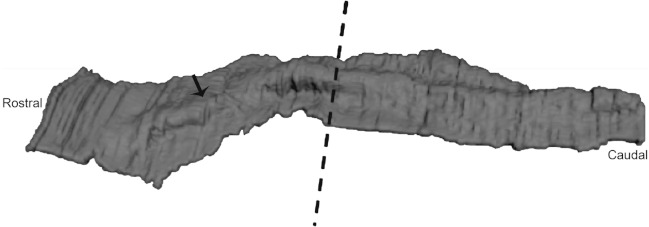
Three-dimensional reconstruction of histological sections of the developing dolphin maxillary arch (LACM 94750). We found that the oral epithelium was thickened but no individual teeth were distinguishable at Carnegie stage 17 suggesting that this stage is comparable in dental development to mouse E10.5-11.5. The arrow points to the thickened ridge. The dashed line indicates the separation of the anterior and posterior part of the jaw into equal portions. 3D reconstruction was generated by taking high resolution photographs of unprocessed serial sections (approximately 737 sections), tracing the oral epithelium, followed by aligning and stacking each tracing in Amira (Amira 4.0, Visage Imaging).

**Figure 5:**
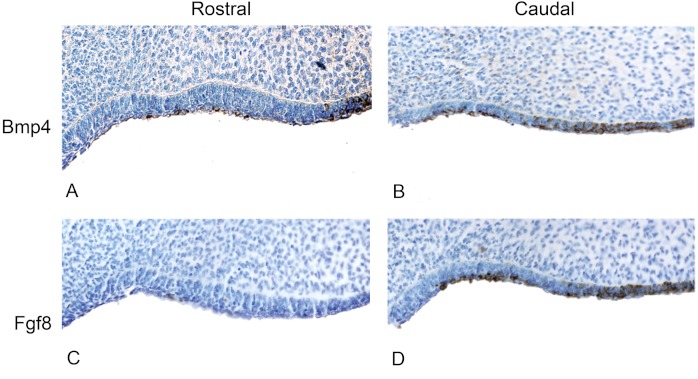
The presence of BMP4 and FGF8 protein during the oral epithelial thickening stage using immunohistochemistry on dolphin embryos (LACM 95670). (A) The anterior (rostral) oral epithelium showing BMPp4 presence. (B) The posterior (caudal) oral epithelium also showing BMP4 presence. (C) The anterior (rostral) oral epithelium showing no FGF8 presence. (D) The posterior (caudal) oral epithelium showing FGF8 presence.

## Results

### Expression of BMP4 and FGF8 in pigs

We investigated pig dental development to test the hypothesis that a representative artiodactyl with a generalized dentition shares similar gene expression during early dental patterning with mice and shrews. *In situ* hybridization experiments were conducted on E20-E24 day old pig embryos that correspond with mouse embryonic day E10.5-E11.5 dental development, as evidenced by the thickening of the oral epithelium. Similar to mice, BMP4 was found in the more rostral regions of the jaw, while FGF8 was found in the more caudal region. These gene expression patterns in early pig tooth development closely match the pattern found in both mice (Fig. 3) and shrews, and presence of protein product was confirmed using immunohistochemistry (data not shown, specimens listed in Table 1).

### BMP4 and FGF8 localization in dolphins

We studied protein localization in embryos of the pantropical spotted dolphin *Stenella attenuata* Carnegie stage 17, a stage at which the dental lamina is prominent (Fig. 4). Consistent with mice and pigs, the rostral region of the dolphin jaw showed immunohistochemical staining of BMP4, but not FGF8 (Fig. 5), whereas FGF8 was limited to the caudal region of the jaw. Surprisingly, BMP4 staining also occurred in the caudal region of the dolphin jaw (Fig. 5). This is contrary to the pattern found in mouse and pig and suggests a potential developmental basis for the differences in tooth shape, complexity and number found in toothed cetaceans.

### Cetacean tooth evolution

**Figure 6:**
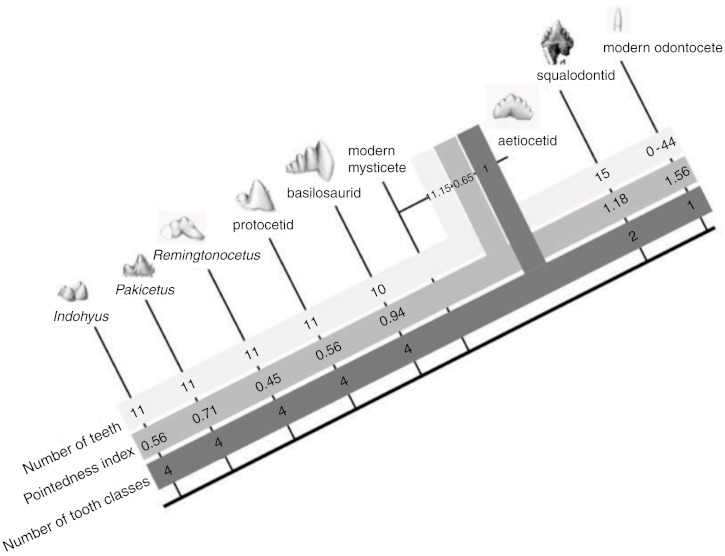
The evolution of cetacean tooth morphology. Grey bars indicate, respectively, number of teeth per jaw quadrant, pointedness and number of tooth classes. Modern mysticete dental patterns are unknown due to their tooth buds resorbing before eruption.

The fossil record documents changes in cetacean dental morphology in detail (Fig. 6). The basal Eocene artiodactyl relative of whales (the raoellid *Indohyus*) and early cetaceans (represented by Eocene *Pakicetus*, *Remingtonocetus*, and a protocetid) retained the basal artiodactyl dental formula of three incisors, one canine, four premolars and three molars per jaw quadrant (Thewissen & Heyning, 2007; Cooper, Thewissen & Hussain, 2009). However, even the first fossil whales (pakicetids) already displayed a loss of molar cusps as compared to their sister group (*Indohyus* in Fig. 6, (Thewissen et al., 2001)). Later cetaceans exhibit varied molar morphologies (Uhen, 2009). Eocene remingtonocetids and basilosaurids have premolars and molars with extra cusps positioned only in the mesio-distal plane. These additional cusps are not homologous to the cusps on tribosphenic molars of mammals.

Throughout the Eocene, molar complexity changes, with the families Pakicetidae, Ambulocetidae, Remingtonocetidae, Protocetidae, and Basilosauridae. Then, at the beginning of the Oligocene, 34 million year ago, early mysticetes and odontocetes increase the numbers of teeth and reduce crown complexity. Modern odontocetes are highly variable and possess between 0 and 50 single-cusped teeth per quadrant, while living mysticetes do not erupt any teeth. In a theoretical model of dental evolution, Salazar-Ciudad & Jernvall (2010) hypothesize that homodonty is correlated with tooth shape and that as individual teeth become more pointed, the tooth row becomes more uniform. Indeed, our data on cetacean cusp pointedness match their model predictions as cusp pointedness increases with homodonty in odontocete evolution (Table 2, Fig. 6). Interestingly, however, this increase in pointedness is not unidirectional across the Eocene, as tooth-pointedness in early archaeocetes (pakicetids) is increased compared to that of clades closer to the cetacean crown group.

**Table 2:** Pointedness index through cetacean evolution. Acronyms – AMP, Ashoro Museum of Paleontology, Ashoro, Japan; IITR-SB, Indian Institute of Technology, Roorkee, India; RR, Obergfell-Rangarao Trust for Geosciences, Dehradun, India.

Taxon	Reference/specimen number	Pointedness index
Raoellidae	ref. Thewissen & Heyning (2007)	0.56
	sp. # RR207, RR211, RR330	
Pakicetidae	ref. Thewissen & Hussain (1998)	0.71
Remingtonocetidae	ref. Thewissen et al. (2001)	0.45
Protocetidae	sp. # IITR-SB 3189	0.56
Basilosauridae	ref. Uhen (2004)	0.94
Aetiocetidae	ref. Barnes, Inuzuka & Hasegawa (1995)	0.65
	sp. # AMP 12	
Modern Mysticeti	ref. Karlsen (1962)	-
Squalodontidae	ref. Van Beneden (1864)	1.18
Modern Odontoceti	ref. Miyazaki (2002)	1.56

## Discussion

### Fossil evidence of developmental integration in cetacean dental patterns

Fossil tooth morphology in early cetaceans supports our hypothesis that tooth number and shape are correlated throughout cetacean evolution. The observed qualitative association is supported by an initial phylogenetic correlation (*r* = 0.72, *p* = 0.043) between independent contrasts for tooth number and pointedness across these groups. These independent contrasts were generated in the PDAP:PDTREE module of Mesquite 2.75 using the data and phylogeny shown in Fig. 6 (branch lengths were set to 1.0 and modern odontocete tooth number to 44 per quadrant to correspond with the pointedness measurement of *Stenella*).

We propose that as cetaceans became more aquatic and began feeding without precise occlusion, the developmental mechanisms that determine tooth class and number underwent a period of experimentation. This can be seen in basilosaurids, aetiocetids and squalodontids: tooth number increase along with crown complexity in these groups (Fig. 6). We interpret this to mean that there was a phase of experimentation with dental shape (and perhaps *Bmp4* expression in the caudal oral epithelium) that preceded the origin of homodonty and the loss of tooth classes in modern cetaceans. At the beginning of that stage, cuspal occlusion was still retained in cetaceans, as indicated by their wear facets, at least in basilosaurids (Thewissen & Heyning, 2007). After that, fundamental shifts increasing pointedness, homodonty and tooth number occur in the Oligocene in early odontocetes (squalodontids) and mysticetes (aetiocetids). The coordinated appearance of these dental changes in the paleontological record suggests integration among elements that may be directed by a single (or relatively fewer) evolutionary change(s) in dental development.

### BMP4 distal expression reflects the unique dentition of the dolphins

The BMP4 domain in embryonic dolphins is expanded into the caudal oral epithelium, and overlaps with the FGF8 expressing field. We propose that this evolutionary change is analogous to laboratory-based manipulations of BMP4 expression. For example, Sharpe (1995) hypothesized that the overlapping incisor and molar expression fields would produce a canine, morphologically similar to dolphin teeth, and experimental studies in mice provide direct evidence that the presence of BMP4 and FGF8 protein in the caudal region of the oral epithelium leads to the formation of ectopic, simple-crowned teeth (Kassai et al., 2005; Murashima-Suginami et al., 2007; Munne et al., 2009). Furthermore, modulation of BMP4 concentration has been implicated in the formation of tooth placode size, leading to changes in tooth number, size and complexity (Munne et al., 2010). We hypothesize that the extension of BMP4 signaling caudally would lead to both an increase in tooth number and the conical, simplified crown shapes found in many extant and fossil cetaceans.

### *Bmp4* and *Fgf8* expression establish the primitive mammalian dental pattern

Non-mammalian amniotes, who do not require precise occlusion, usually have many single-cusped, conical teeth. In homodont bony fish, *Bmp4* and *Fgf8* are co-expressed throughout the oral epithelium, although *Fgf8* does not play a role in early tooth development in these vertebrates (Streelman & Albertson, 2006; Fraser, Bloomquist & Streelman, 2008). Furthermore, modification of a trans-acting molecule on BMP4 has been postulated to determine tooth number and cusp number in cichlid fish (Albertson & Kocher, 2006).

**Figure 7:**
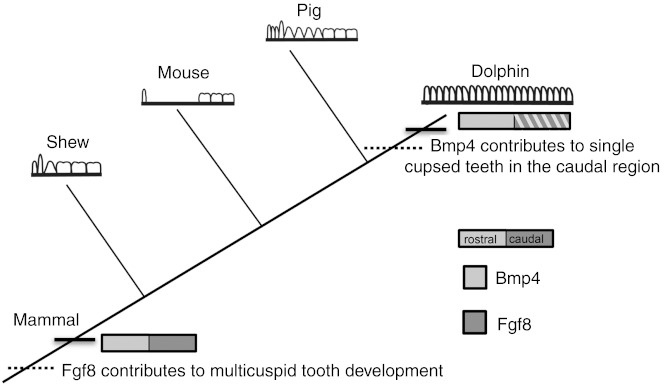
Mammalian tooth class development. Cladogram showing known expression patterns of *Bmp4* and *Fgf8* across investigated mammals. Dotted lines indicate hypothesized major changes in dental patterning.

At the origin of mammals, the evolution of novel tooth shapes, such as multicuspid teeth, may have occurred partly by modulation of *Bmp* expression (Ohazama et al., 2010a) (Fig. 7). These novel tooth shapes are related to functional occlusion and stand at the root of mammalian high metabolic rate and diversity. The similar expression pattern of *Fgf8* and *Bmp4* in the developing dental lamina across mice, shrews and pigs suggests that this pattern is constrained across mammals that have molars and incisors, regardless of the details in dental pattern (Fig. 7). This constraint is most likely due to the need for mammals to maintain precise occlusion facilitating the mechanical breakdown of food during mastication as a key innovation underlying their elevated metabolic rate. When precise occlusion is lost in mammals such as seals, armadillos and cetaceans, developmental mechanisms are less constrained allowing for evolutionary experimentation in cusp pattern and tooth number.

## Conclusions

Macroevolutionary changes, such as those characterizing cetacean dental anatomy, are driven by changes in the regulation of gene expression domains during early development (Hall, 1999; Carroll, Grenier & Weatherbee, 2001). Little is known about cetacean dental embryology (Karlsen, 1962; Míšek et al., 1996) and even less is known about the developmental mechanisms that drove the evolution of the cetacean body plan (Richardson & Oelschläger, 2002; Thewissen et al., 2006). The evolution of the cetacean dentition provides a macroevolutionary case study that offers a unique opportunity to combine paleontological data with a modern understanding of genetic control of early dental development in mammalian model taxa. It leads us to hypothesize about the molecular changes that occur throughout cetacean and mammalian evolution. We propose that a primitive pattern of rostral *Bmp4* and distal *Fgf8* expression across mammals was modified during cetacean evolution allowing a caudal expansion of *Bmp4* expression in the jaw that led to the extra teeth with simple, similar crowns seen in living toothed whales.
